# High-intensity focused ultrasound as a combined approach for the treatment of recurrent low-grade endometrial stromal sarcoma: a case report and literature review

**DOI:** 10.3389/fonc.2025.1551792

**Published:** 2025-11-24

**Authors:** Huihui Chen, Xiaonan Shang, Yue Shen, Huajing Huang, Zebo Jiang, Qingyi Wang, Zhixing Cao, Peiyu Yan, Suying Xiao, Liangyu Chen, Donghui Huang, Min Kang

**Affiliations:** 1 Faculty of Chinese Medicine, Macau University of Science and Technology, Macao, Macao SAR, China; 2 Zhuhai Hospital of Integrated Traditional Chinese Western Medicine, Zhuhai, Guangdong, China; 3 Northeastern University, Boston, MA, United States; 4 Zhuhai People’s Hospital, Zhuhai, Guangdong, China; 5 State Key Laboratory of Quality Research in Chinese Medicines, Macao, Macao SAR, China; 6 Macau University of Science and Technology Zhuhai MUST Science and Technology Research Institute, Macao, Macao SAR, China

**Keywords:** LGESS, HIFU, tumor recurrence, combination therapy, case report

## Abstract

**Background:**

Surgery is the primary treatment for Endometrial Stromal Sarcoma (ESS), however, a substantial proportion of patients with ESS experience recurrence or metastasis. Currently, surgery and local ablation are the main treatments for recurrent ESS followed by chemotherapy, radiotherapy, immunotherapy, targeted therapy, and anti-estrogen therapy. Surgery and local ablation are invasive treatments and may carry risks such as intestinal damage and the risk of massive bleeding from tumor rupture. For patients who refuse or are unable to undergo surgery and local ablation, conservative treatment is not effective, and there is currently no definitive effective non-invasive or combined treatment plan.

**Case presentation:**

This report presents a case of a patient with recurrent endometrial stromal sarcoma who refused surgical and local ablation treatments. After receiving HIFU treatment combined with chemotherapy, the progression of the tumor was effectively inhibited, the tumor volume significantly reduced, and liver function was restored during the HIFU period, providing an opportunity for chemotherapy.

**Conclusions:**

HIFU combined with chemotherapy may provide a new treatment strategy for patients with recurrent, metastatic endometrial stromal sarcoma, or those who are unsuitable for surgery, local ablation, or those with poor baseline status unable to tolerate intensive chemotherapy.

## Introduction

ESS is an invasive tumor originating from endometrial stromal cells. The cells resemble proliferative phase endometrial stromal cells, manifesting as infiltrative growth, with or without lymphovascular invasion. It accounts for approximately 0.2-1% of uterine malignancies and 6-20% of uterine sarcomas (1–3). According to the WHO (2020 edition) classification of gynecological malignancies, ESS is divided into Low-Grade Endometrial Stromal Sarcoma (LGESS) and High-Grade Endometrial Stromal Sarcoma (HGESS) (4).

Due to the lack of specific clinical and radiographic manifestations, ESS is easily misdiagnosed as uterine fibroids or adenomyosis with similar symptoms (5). Therefore, a thorough evaluation must be performed on rapidly enlarging fibroid masses before surgery. High-grade stromal sarcoma carries a poor prognosis, especially when diagnosis is delayed or presented with advanced stages (6). LGESS is typically discovered during pathological examination of hysterectomy specimens (7). LGESS is a relatively indolent tumor with a good overall survival rate, but it is characterized by multiple or late recurrences (3, 8). Recurrence is more common in the pelvic and abdominal cavities, and less common in the lungs and vagina. Due to its indolent course, distant recurrence is more frequently seen in clinical practice, necessitating long-term follow-up, hence there is less research on the prognosis of recurrent LGESS (9).

Currently, hysterectomy and bilateral salpingo-oophorectomy are the first-line treatments for ESS. However, approximately 30%-50% of ESS patients experience recurrence or metastasis (10). At present, surgical treatment, anti-estrogen therapy, chemotherapy, radiotherapy, and targeted drug therapy are used to treat recurrent or metastatic ESS. However, due to the different pathological characteristics and fewer cases, there is not enough research and data, and the treatment plan for recurrent metastatic ESS is still not clearly unified.

In terms of examinations and follow-up, MRI differentiates uterine fibroids from sarcomas through its superior soft-tissue resolution, while monitoring tumor volume changes and therapeutic effects. PET-CT precisely identifies metastases or recurrent lesions, yet its phased utilization is prioritized in clinical practice due to cost and procedural constraints. MRI serving as the foundational modality, while PET-CT provides targeted assistance.

We report a case of recurrent low-grade endometrial stromal sarcoma with multiple pelvic metastases and right sacral bone metastasis. The patient had a short-term recurrence after surgery and underwent multiple rounds of combined radiochemotherapy and regular follow-up. Three years later, the patient relapsed again. After hospital evaluation, the patient was unwilling to undergo a second surgery due to concerns about surgical risks. The patient then received three cycles of chemotherapy. After chemotherapy, the patient developed abnormal liver function. After discussion by the doctors, the treatment plan was changed to HIFU and chemotherapy. This effectively inhibited tumor progression with significant results.

## Case report

**Figure 1:**
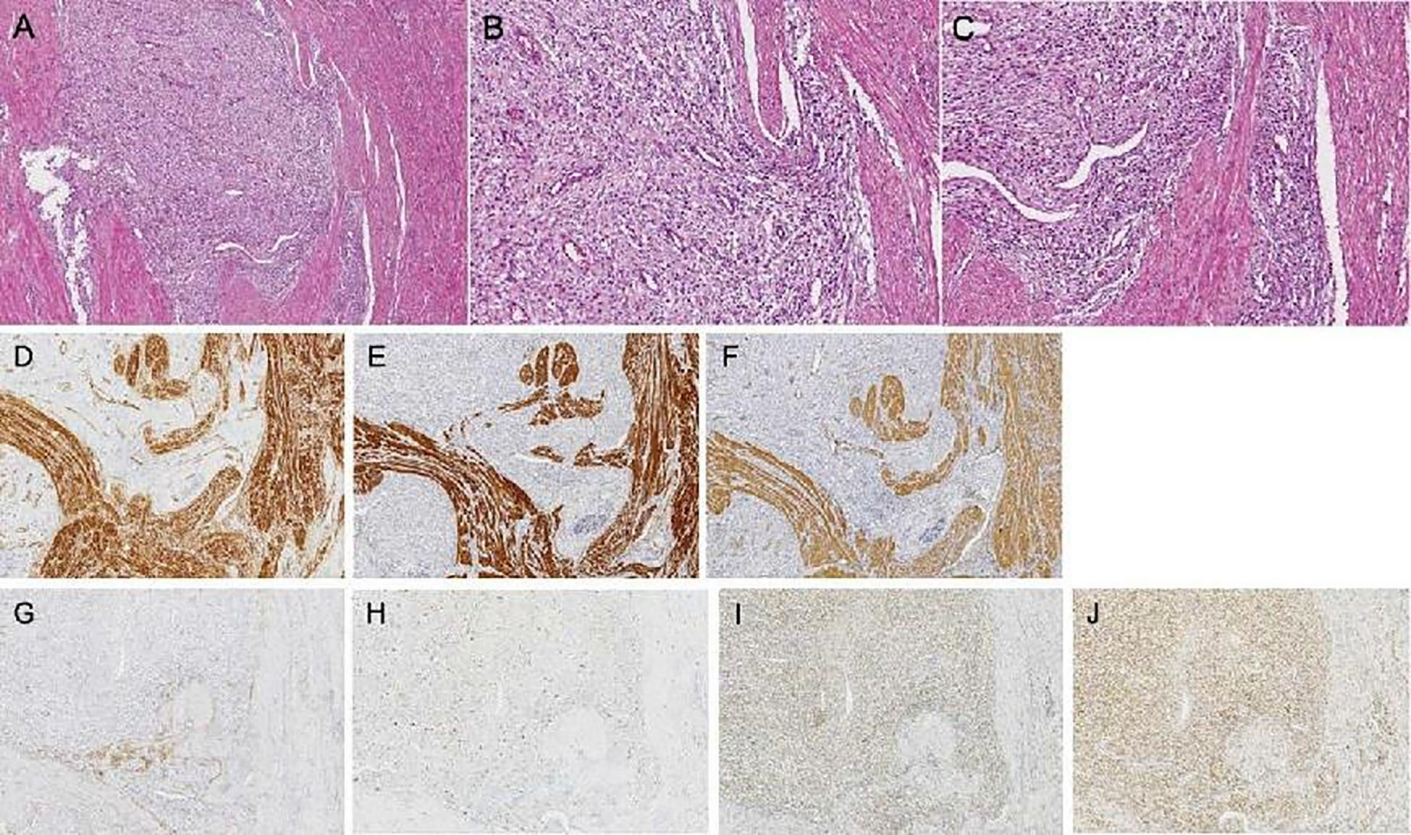
Tumor histopathology images [**(A-C)** Hematoxylin-eosin staining; magnification: **(A)** 4×; **(B, C)** 10×]. Main immunohistochemical staining results for low-grade endometrial stromal sarcoma [**(D–J)** 4×]. **(D)** Caldesmon; **(E)** Desmin; **(F)** SMA; **(G)** CD10; **(H)** Ki-67; **(I)** ER; **(J)** PR.

The patient is a 28-year-old unmarried and nulliparous female with no family history of malignancy and no prior gynecological disorders or estrogen-related medication use. She presented to the hospital in November 2019 with progressively worsening dysmenorrhea for one year and menorrhagia for six months. Gynecological ultrasound and abdominal CT indicated an enlarged uterus, suggesting uterine fibroids. On November 21, 2019, she underwent laparoscopic exploration. During the operation, a tumor approximately 9*9*8cm in size was seen on the posterior wall of the uterus, and another tumor approximately 5×4cm in size was seen on the lower segment of the posterior wall of the uterus. The intraoperative frozen pathology diagnosis was a mesenchymal malignant tumor. With the consent of the family, the operation was changed to total hysterectomy, bilateral adnexectomy, and omentectomy. Postoperative pathology and immunohistochemistry indicated low-grade endometrial stromal sarcoma with transformation to high-grade, local necrosis, enlarged and round nuclei, invasion of the uterine myometrium, involvement of the endometrium and serosal layer, tumor invasion seen in the blood vessels, no tumor invasion seen in the nerves, and no tumor seen in the bilateral adnexa and omentum (**Figure 1**). The postoperative pathological stage was stage IB. After the operation, she underwent three rounds of intraperitoneal hyperthermic perfusion therapy (cisplatin 110mg).

On December 17, 2019, the patient’s follow-up 18F-FDG PET/CT (18F-fluorodeoxyglucose positron emission computed tomography/computed tomography) showed thickening of vaginal soft tissue with increased glucose metabolism, multiple pelvic lymph nodes with increased glucose metabolism, suggesting metastasis. The right side of the sacrum showed slightly increased bone density with increased glucose metabolism, suggesting possible metastasis. On December 23, 2019, an enhanced whole abdomen MR suggested a nodular lesion on the left margin of the vaginal stump, highly suspicious of tumor; multiple lymph nodes near bilateral iliac vessels, on both sides of the pelvis, and in the pre-sacral space, lymph node metastasis could not be excluded. The preliminary diagnosis was “vaginal recurrence of endometrial stromal sarcoma and sacral metastasis”. From January 9 to January 20, 2020, the patient underwent VMAT radiotherapy (dose: GTVnd 6000cGy, CTV4500cGy). From February 28 to March 12, 2020, the patient underwent 4 sessions of brachytherapy (dose: 28Gy/4f, cisplatin as a radiosensitizer). On January 9 and January 20, 2020, she received concurrent chemotherapy with cisplatin (dose: 25mg, d1-4). On February 21, 2020, she accepted chemotherapy with cisplatin (100mg) and nivolumab (200mg) and regorafenib capsules (20mg, Bid). On March 19, 2020, she accepted a cycle of chemotherapy with paclitaxel (300mg) and lobaplatin (150mg) and nivolumab (200mg).From April 15 to June 3, 2020, she continued to receive 3 cycles of chemotherapy with lobaplatin (150mg) and paclitaxel (330mg) and bevacizumab (350mg). On April 15, 2020, she underwent a biopsy of the vaginal lesion, and the pathology results indicated: (vaginal orifice nodule) no endometrial stromal sarcoma seen. Serial MRI scans performed every three months between January and June 2020 revealed no abnormalities. Following the completion of chemotherapy, the patient underwent PET/CT scans every six months, with no significant abnormalities detected in the results.

On August 18, 2023, the patient experienced pain in the lower left abdomen, which gradually worsened, accompanied by left-sided back pain and fever. Outpatient ultrasound examination of the urinary system suggested: dilation of the upper segment of the left ureter with hydronephrosis of the left kidney, and a hypoechoic mass behind the bladder, measuring approximately 88×74×88mm, with clear boundaries and uneven internal echo. On August 24, 2023, a PET/CT scan showed a mass of approximately 87×83×90mm at the vaginal stump, suggesting a possible recurrence of the tumor.

**Figure 2:**
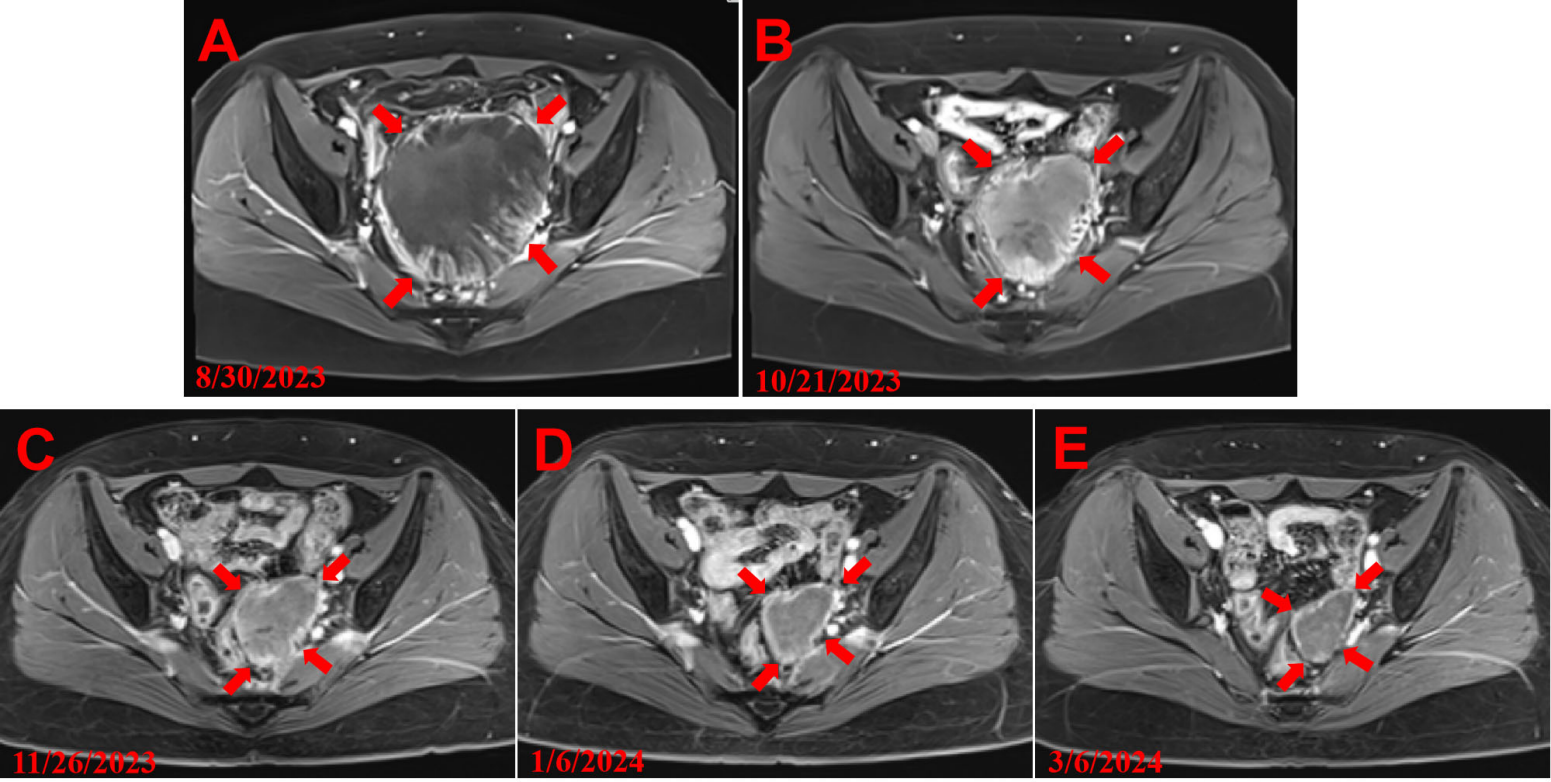
MRI images of the patient. **(A)** Tumor volume size of the patient’s first MRI after recurrence. **(B)** Tumor volume size before the first HIFU treatment after three courses of chemotherapy with TC regimen. **(C)** Tumor volume size after first HIFU treatment. **(D)** Tumor volume size after the fourth course of TC regimen chemotherapy. **(E)** Tumor volume size after the second HIFU treatment.

The patient was admitted to the hospital for treatment on August 28, 2023, and underwent enhanced abdominal MR and urinary CTU examinations. The MR enhancement suggested an abnormal signal in the pelvic cavity, measuring approximately 99mm×88mm×116mm, suggesting local tumor recurrence, possibly involving the rectum, colon, bladder, and left ureter. After pelvic metastasis, the patient’s primary symptoms included left-sided lumbar soreness, abdominal distension, and lower abdominal pain. Physical examination revealed a pelvic mass measuring approximately 9 cm×8 cm on triple examination, with a firm consistency, poor mobility, no significant tenderness, and no percussion tenderness over the sacrococcygeal region. After multidisciplinary consultation, the patient was informed of the high risk of surgery, including potential intestinal and bladder injury, and the possibility of performing intestinal and renal fistula surgery, ablation therapy may carry risks of tumor rupture and bleeding, and injury to the intestines and bladder. The patient strongly refused surgery and ablation therapy, requesting conservative treatment. After ruling out contraindications to chemotherapy, the patient underwent three cycles of systemic chemotherapy with the TC regimen (paclitaxel injection 260mg + carboplatin injection 500mg) on September 5, September 26, and October 23, 2023. The tumor size decreased from 99mm×88mm×116mm to 69.2mm×57.8mm×75.4mm (**Figures 2A, B**).

On November 12, 2023, the patient’s liver function showed significant abnormalities (Alanine transaminase (ALT): 47.7 U/L;Aspartic amino transferase (AST): 32.8 U/L;CTCAE version 5.0: Grade 1 hepatotoxicity) and she could not receive the fourth cycle of chemotherapy as scheduled. After discussion and with the patient’s consent, the treatment plan was changed to HIFU treatment and liver protection treatment, waiting for the opportunity for chemotherapy. From November 13 to November 24, 2023, the patient underwent nine intermittent HIFU treatments, after which the blood flow in the pelvic tumor significantly decreased. During this period, the patient was given liver protection treatment (silymarin capsules 140mg, bid, orally), and glutathione (1.2g, qd, intravenous infusion). On December 12, 2023, the patient’s liver function recovered, and she underwent the fourth cycle of systemic chemotherapy with the TC regimen (paclitaxel injection 260mg + carboplatin injection 600mg). On January 5, 2024, the patient’s liver function was abnormal again (Alanine transaminase (ALT): 118.6 U/L;Aspartic amino transferase (AST):41.6 U/L;CTCAE version 5.0: Grade 1 hepatotoxicity) and she could not receive the fifth cycle of chemotherapy as scheduled. From January 8 to January 16, 2024, the patient received eight intermittent HIFU treatments, and liver protection treatment was continued during the treatment period. On February 2, 2024, the patient’s liver function recovered, and she underwent the fifth cycle of systemic chemotherapy with the TC regimen (paclitaxel injection 270mg + carboplatin injection 780mg). After 17 HIFU treatments combined with chemotherapy, the patient’s lesion decreased from 69.2mm×57.8mm×75.4mm to 43mm×33mm×45mm (**Figures 2C–E**). The scattered small nodules in the original pelvic cavity disappeared, the dilation of the upper segment of the original left ureter improved significantly, the turbidity of the fat space in the original pelvic cavity and the pelvic effusion disappeared. The edema of the left piriformis muscle significantly improved. The level of tumor markers gradually decreased and tended to stabilize. The patient’s abdominal pain and bloating symptoms disappeared, and she had no other discomfort. On March 8, 2024, she underwent the sixth cycle of systemic chemotherapy with the TC regimen (paclitaxel injection 270mg + carboplatin injection 650mg).

**Figure 3:**
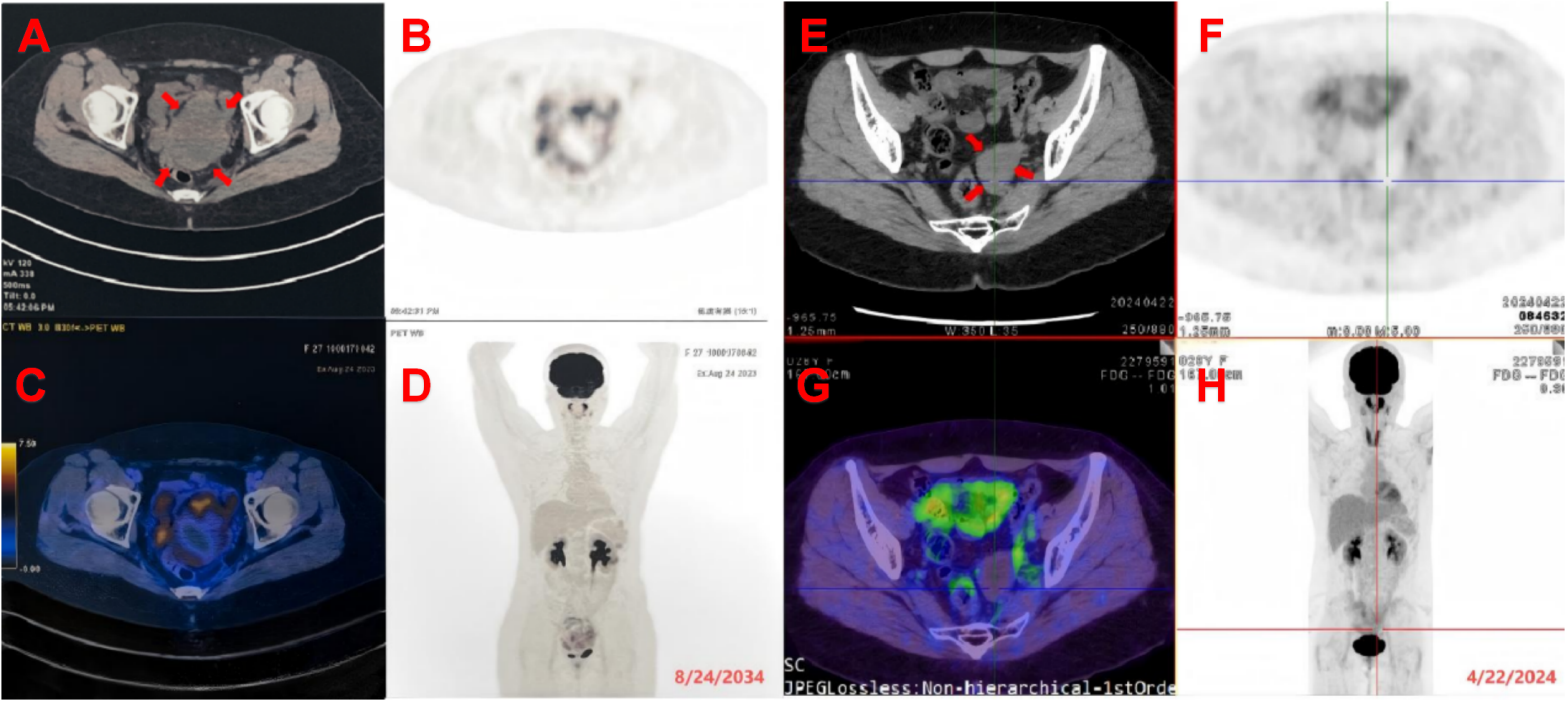
**(A–D)** Pre-treatment PET/CT: A pelvic soft tissue mass demonstrates heterogeneously intense radiotracer uptake with a maximum standardized uptake value (SUVmax) of approximately 7.0. The lesion measures approximately 8.7 × 8.3 × 9.0 cm, showing internal necrotic components. The mass invades the vaginal stump and exhibits ill-defined borders with the rectum and the pelvic segment of the left ureter. **(E–H)** Post-treatment PET/CT after 6 courses of chemotherapy and 2 sessions of HIFU therapy.A hypodense lesion is noted in the left pelvis, measuring approximately 4.7 cm × 3.1 cm. It demonstrates ill-defined borders with the vaginal stump and has an SUVmax of 2.8.

**Figure 4:**
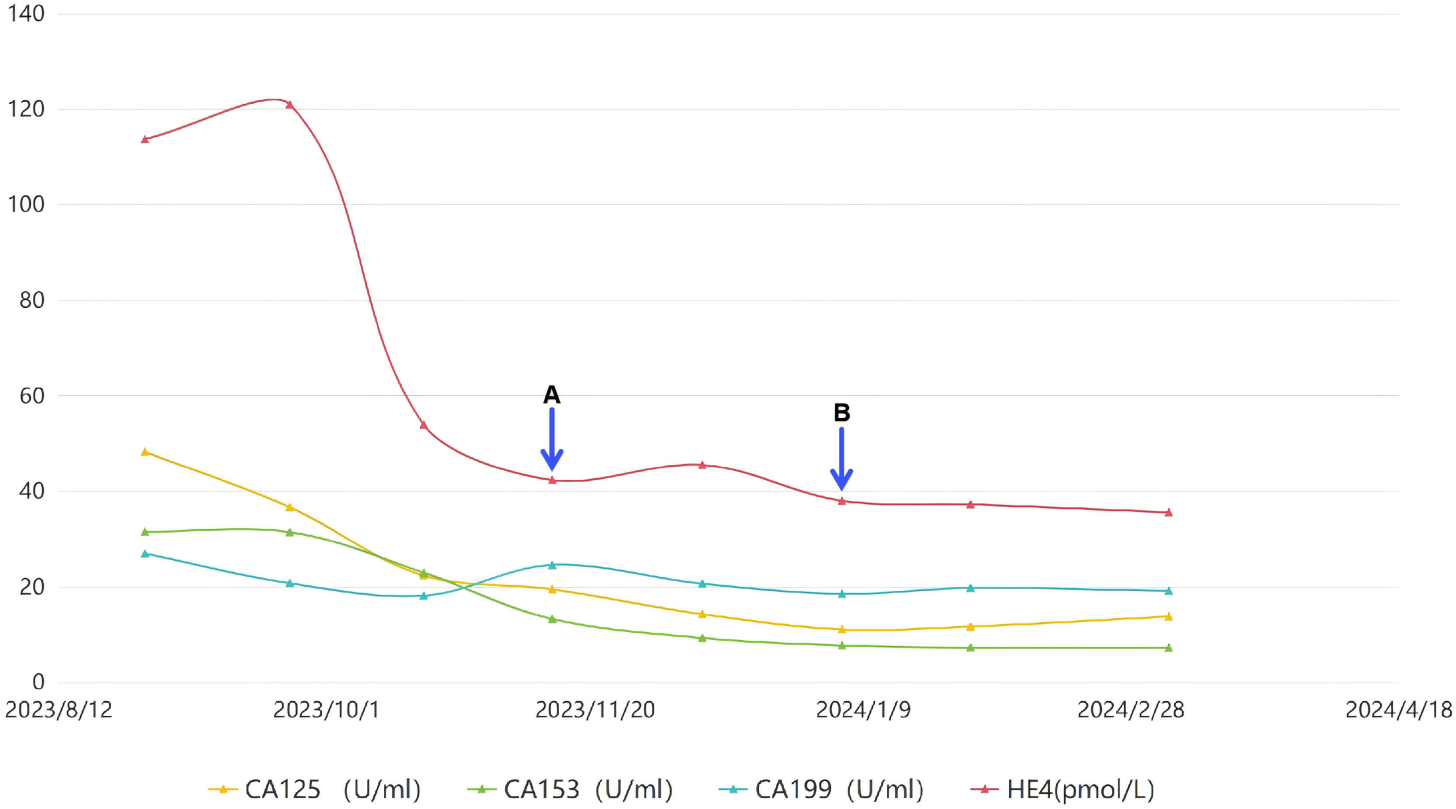
The trend chart of various tumor indicators. **(A)** First HIFU treatment; **(B)** Second HIFU treatment.

**Figure 5:**
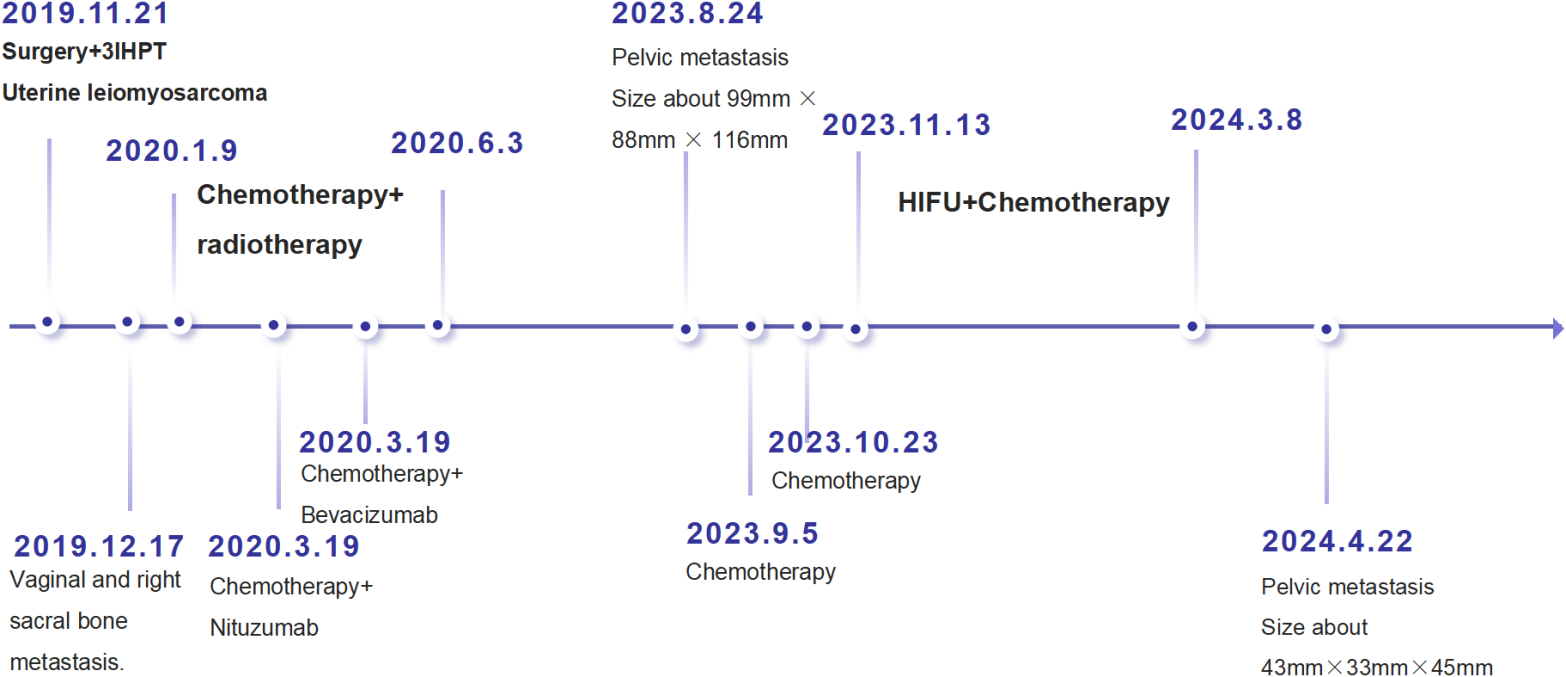
Time-event axis.

On April 22, 2024, a PET/CT scan suggested that the blood flow signal around the patient’s pelvic mass had significantly decreased, the mass had basically shown changes after HIFU treatment (**Figure 3**), and the patient’s tumor markers (**Figure 4**) had steadily decreased and trended toward stabilization. The treatment effect was satisfactory. The patient was advised to undergo surgical treatment, but she still refused. The benefit of immunotherapy for the patient was not evident at present. The patient requested regular follow-up, and there were no new lesions at present. It is recommended to continue regular HIFU maintenance treatment in the future. The patient is currently under continued follow-up observation. The disease timeline is shown in **Figure 5**.

## Methods

In this case, the patient used the yLab Class C Ultrasound Diagnostic System (Shenzhen Baisheng Medical Equipment Co., Ltd) and the HIFUNIT9000 Focused Ultrasound Tumor Ablation Machine (Shanghai Aishen Technology Development Co., Ltd). The system consists of a main unit, motor system, control console, monitoring system, power supply, and water treatment system.

Pre-treatment preparation: The patient was instructed to abstain from high-protein food the day before the treatment. Prior to the treatment, the patient was asked to retain a small amount of urine to fill the bladder. Lactulose oral solution (Beijing Hanmei, 100ml/bottle) was administered for bowel preparation, and parecoxib sodium (Dynastat) was administered via intramuscular injection for analgesia.

During the treatment, phloroglucinol injection was administered intravenously. The patient was positioned supine, and the machine located the pelvic tumor. Throughout the procedure, the treatment intensity and duration were adjusted according to the patient’s tolerance and the grayscale changes displayed on the ultrasound.

HIFU is a non-invasive therapeutic technique that does not require anesthesia, has no incisions, no radiation, and has a quick recovery time. It is primarily used for solid tumors that can be observed under ultrasound, such as adenomyosis, uterine fibroids, osteosarcoma, most primary and metastatic liver tumors, etc.

**Figure 6:**
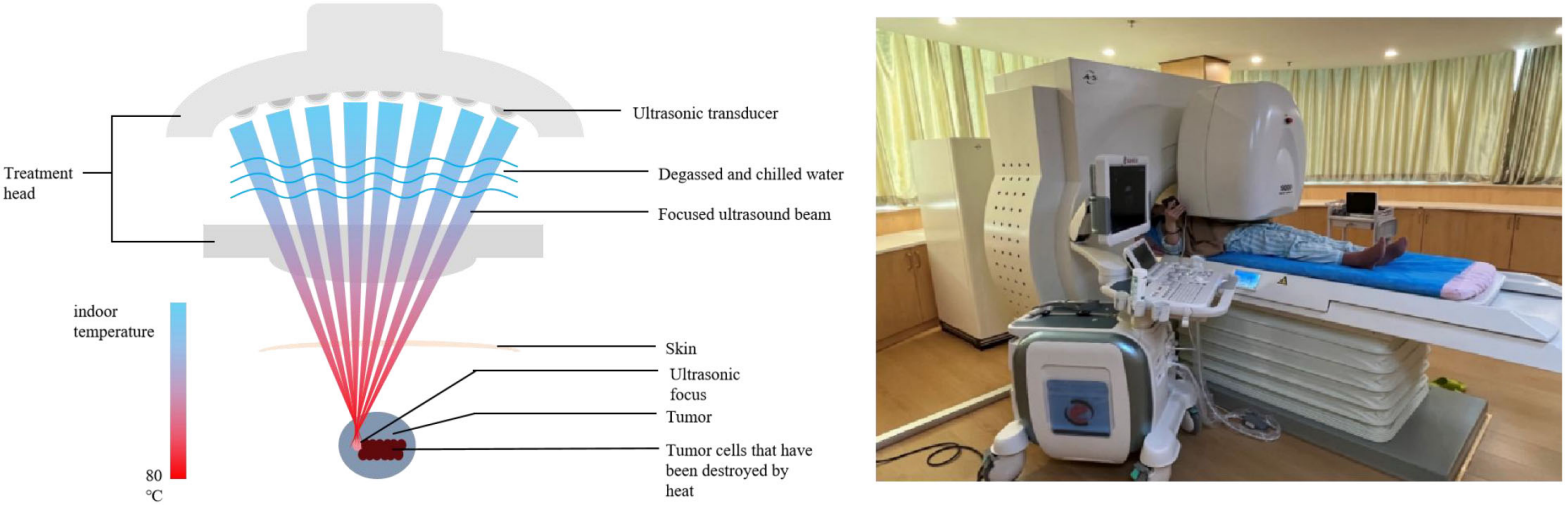
Schematic diagram of HIFU operation principle.

The principle of HIFU treatment involves precise positioning and outlining of the tumor under ultrasound, scanning point by point and layer by layer according to the shape of the tumor. Utilizing the penetrative and focusing properties of ultrasound waves, the waves emitted from outside the body are focused on the pathological tissue inside the body. Through thermal effects, mechanical effects, and cavitation effects, the temperature of the pathological tissue rises instantly to 60-100°C, causing instantaneous irreversible cell death and coagulative necrosis of the tumor tissue. HIFU therapy achieves precise targeted ablation through real-time ultrasound imaging guidance. A 3.5–5 MHz dual-mode transducer enables simultaneous visualization of anatomical structures and blood flow distribution. During treatment, gray-scale ultrasound images are acquired at 5-minute intervals, monitoring echo intensity enhancement in the target area (indicative of coagulative necrosis). Initial acoustic intensity is set at 300–500 W/cm², with dynamic adjustments to pulse frequency (0.8–1.2 MHz) and duty cycle (30–50%) based on real-time thermal curves (target temperature 55–65°C). Should acoustic pathway deviation occur (e.g., due to bowel gas interference), immediate treatment suspension and refocalization of the acoustic energy are implemented (**Figure 6**).

In the assessment of patient adaptability, it is mainly based on the evaluation of subjective symptoms such as lumbosacral pain, abdominal pain, lower - limb neuralgia, and local skin burning during the patient’s treatment. If the pain and skin burning are obvious, the energy intensity should be reduced or the treatment should be suspended. Regarding the efficacy assessment, there are currently no precise treatment standards and data. Our clinical experience mainly relies on ultrasound examinations. A better treatment effect is indicated when, in comparison before and after treatment, the area of enhanced echo of the mass under ultrasound exceeds 90%, the closure rate of small blood vessels exceeds 70%, and the reduction rate of blood - flow signals in local thick blood vessels exceeds 50%.

## Discussion

ESS is a relatively rare gynecological malignancy. The treatment of recurrent ESS remains a challenge. The 5-year survival rate for patients with stage I and II low-grade ESS reaches 90%, while for patients with stage III and IV, it is about 50% (9). Previous studies have reported recurrence rates of LGESS ranging from 10% to 76%, which may be due to its characteristic of recurrence over 5 years, resulting in a large difference in recurrence rates (11). At present, the main treatment option for endometrial stromal sarcoma is surgery, supplemented by chemotherapy, radiotherapy, anti-estrogen therapy, etc. Due to the many adverse reactions of radiotherapy and chemotherapy and the inability to continue, for patients who cannot undergo surgery, ablation therapy can be chosen. However, the ablation process requires puncture, which may damage surrounding organs such as the intestines and bladder. The puncture process may lead to the risk of tumor rupture, bleeding, and tumor dissemination and metastasis. Therefore, the treatment of recurrent ESS remains a significant challenge.

In this case, the patient was diagnosed with LGESS and experienced rapid recurrence shortly after surgery. After multiple rounds of combined radiotherapy and chemotherapy, the condition stabilized and regular follow-up examinations were scheduled. In August 2023, the tumor recurred again, measuring approximately 10cm, with multiple pelvic metastases and right sacral bone metastasis. The tumor was suspected of locally recurring and possibly invading the rectum, colon, bladder, and left ureter. Unable to accept the risks of surgery, the patient strongly refused surgical intervention and underwent chemotherapy. After three cycles of chemotherapy, the tumor size decreased from 99mm×88mm×116mm to 69.2mm×57.8mm×75.4mm. However, due to abnormal liver function, the fourth cycle of chemotherapy could not be administered. After evaluation and discussion, HIFU treatment was added, liver protection treatment was administered during this period, and the timing for chemotherapy was awaited. After 17 sessions of HIFU treatment combined with systemic chemotherapy, the tumor size reduced from 69.2mm×57.8mm×75.4mm to 43mm×33mm×45mm, no longer compressing the bladder and ureter, the scattered small nodules in the pelvic cavity disappeared, the level of tumor markers gradually decreased and stabilized, and the patient’s abdominal pain and bloating disappeared, significantly improving her quality of life. HIFU treatment during periods when chemotherapy cannot be administered can continuously inhibit tumor progression, preventing tumor enlargement during periods without chemotherapy. Combined liver protection treatment is beneficial for the recovery of liver function, allowing the patient to receive chemotherapy on schedule.

HIFU is a novel non-invasive thermotherapy that can cause coagulative necrosis of tumor tissue. It has the advantages of high repeatability, uniform heat diffusion, virtually painless treatment process, no external injuries, rapid postoperative recovery, and no impact on patient function. It has been proven effective and safe in the treatment of solid tumors such as uterine fibroids, breast cancer, and pancreatic cancer. A prospective study suggested that the effectiveness of HIFU in treating uterine fibroids was higher than surgical treatment, and it was safer (12). MR-HIFU treatment significantly alleviates the clinical symptoms caused by uterine fibroids and effectively reduces the tumor volume (13). In addition, a retrospective review findings of HIFU treatment was more effective than secondary myoma resection, with fewer side effects, longer asymptomatic periods, and lower risk of re-intervention (14). A systematic review study showed that patients with postoperative pathological diagnosis of uterine sarcomas (including LGESS and uterine leiomyosarcoma) do not cause histological dissemination of sarcoma after receiving HIFU treatment (15). HIFU treatment has therapeutic effects on uterine fibroids and sarcomas, and also has good effects in the treatment of other pelvic tumors. Zhong Q,etc (16), retrospectively analyzed 153 patients with cervical cancer residual or recurrent after chemoradiotherapy (CRT) who received HIFU treatment from 2010 to 2021. The results showed that HIFU can significantly reduce the size of residual or recurrent lesions, improve local control rates and survival time, and even elderly or physically poor patients can tolerate it, providing a supplementary treatment method for cervical cancer patients with adverse reactions after chemotherapy. Lei T,etc (17), treated 8 patients with recurrent ovarian cancer or metastatic pelvic tumors with HIFU, and found that the pain relief rate was 60%, short-term quality of life improved, and adverse reactions after treatment were mild. Studies have shown that HIFU treatment of pelvic metastatic tumors or recurrent ovarian cancer is feasible and without serious complications. HIFU treatment is also used in breast cancer and pancreatic cancer. Zulkifli D,etc (18), included nine studies and found that HIFU can induce coagulative necrosis of local breast cancer tumors, with small side effects, good cosmetic effects, and a 5-year disease-free survival rate of more than 90%. A meta-analysis evaluated 19 studies with a total of 939 patients, and the results showed that HIFU treatment combined with drug treatment of pancreatic cancer can relieve patients’ chronic pain, the incidence of adverse events is low, and it can improve the overall survival rate (19). In the treatment of prostate cancer, HIFU treatment also plays a role. Parry MG reported that after 1381 patients with prostate cancer received HIFU treatment, the tumor effectively shrank, and urinary and reproductive functions were preserved, with little impact on the quality of life (20).

HIFU is currently used for pelvic and abdominal solid tumors, and the treatment effect is good, patients with residual or recurrent tumors in the pelvis after radiotherapy and chemotherapy also benefit. These research results provide evidence for us to choose to add HIFU in this case, clinical data also prove that HIFU combined with chemotherapy for the treatment of recurrent low-grade endometrial stromal sarcoma is effective and safe.

During HIFU treatment, different tumor sizes and locations are associated with distinct side effects and limitations. To enhance treatment safety, prior to treatment, it is necessary to improve the patient’s nutritional status, control underlying diseases, and establish psychological expectations. Additionally, multi - modal imaging techniques should be employed to precisely locate the lesion. During the treatment, parameters should be dynamically adjusted based on the tumor size, depth, blood supply characteristics, and the patient’s adverse reactions. This ensures effective ablation of the tumor tissue while minimizing damage to the surrounding normal tissues to the greatest extent. After the treatment, measures should be taken as early as possible to address adverse reactions. Hierarchical interventions should be carried out for common problems such as fever, pain, and skin damage. Meanwhile, psychological counseling should be provided to improve the patient’s treatment experience.

The combined HIFU therapy has gained increasing attention, and changes in immune-related markers and tumor biomarkers may be associated with treatment prognosis. Dong S et al. compared pancreatic cancer patients receiving HIFU-priority versus chemotherapy-priority regimens in combined therapy and found that the HIFU-priority group demonstrated significantly improved overall survival (OS) (HR = 0.38) (21). Additionally, patients with normal CRP and CA125 levels exhibited longer survival. Elevated neutrophil-lymphocyte ratio (NLR) and low lymphocyte-monocyte ratio (LMR) were associated with poor prognosis. Wang R et al. found that patients positive for CD133 and other stem cell markers may benefit from targeted nanocarrier-based therapies combined with HIFU (22). HIFU may enhance chemotherapeutic efficacy by creating a tumor hypoxic environment that activates hypoxia-inducible factors (HIFs), thereby improving the delivery efficiency of chemotherapeutic agents such as doxorubicin. Concurrently, HIFU promotes CD4+/CD8+ lymphocyte infiltration into tumor tissues (23, 24). HIFU activates systemic immune responses by releasing tumor antigens and danger signals, with CD8+ lymphocyte infiltration correlating with regression of distant untreated lesions (24, 25). Patients with higher baseline tumor-infiltrating lymphocyte (TIL) levels are more suitable for HIFU combined with PD-1 inhibitors and chemotherapy (26, 27).

In terms of pathological characteristics, the combination of HIFU and chemotherapy significantly controls the growth of recurrent lesions in mucinous ovarian cancer (28). In advanced gastric cancer (GC) patients, HIFU-priority regimens following neoadjuvant chemotherapy significantly improve OS, particularly in stage III patients (HR = 1.61) (29). Multimodal imaging serves as the gold standard for post-HIFU chemotherapeutic response evaluation, with contrast-enhanced CT/MRI clearly delineating tumor anatomy and Extent of necrosis (30, 31). Molecular ultrasound imaging dynamically monitors tumor vascular characteristics (via the QuanTAV index), predicting treatment sensitivity (32). Translucent texture changes in ultrasound/MRI follow-up of muscularis lesions indicate therapeutic efficacy, whereas residual enhancing foci warrant caution for recurrence (33, 34). Future research should prioritize refining a multi-parameter decision model integrating tumor biomarkers, imaging features, pathological staging, and immunological status to optimize HIFU-chemotherapy combination therapy precision.

The main mechanisms by which HIFU combined with chemotherapy may exert its therapeutic effect are likely related to the following aspects. First, tumor cells are more sensitive to high temperatures than normal cells. HIFU destroys tumor tissue through its thermal effect, inducing apoptosis of tumor cells; the thermal effect can increase tumor blood flow and enhance the permeability of the tumor cell membrane, thereby accelerating the penetration and absorption of chemotherapeutic drugs (21, 35). Second, after HIFU treatment, tumor cells die and cellular components enter the bloodstream. The expression of a large number of tumor antigens in the fragments activates the immune system’s anti-tumor response. Third, some studies suggest that HIFU treatment can change the tumor’s resistance to chemotherapy, increasing the sensitivity of tumor cells to chemotherapeutic drugs (36, 37). The anti-tumor mechanism of HIFU treatment is still under research, especially the impact on the immune system which requires further exploration.

While offering the advantage of non-invasiveness, HIFU possesses significant limitations in clinical application. Its efficacy is constrained by tissue acoustic properties; it cannot effectively penetrate gas-containing organs (e.g., lungs) or bone, limiting its use for tumors in locations like the thorax or intracranial cavity. Furthermore, HIFU application is highly dependent on specific tumor characteristics: size, well-defined margins, and proximity to critical vasculature or nerves. Tumors that are excessively large or unfavorably located pose procedural risks, including potential damage to adjacent structures such as bowel loops or nerves.

Additionally, real-time monitoring during treatment and accurate post-procedural efficacy assessment remain challenging. The inability to obtain tissue samples for histopathological confirmation necessitates reliance on post-treatment imaging follow-up for evaluating response. Procedural success heavily depends on operator expertise, resulting in a steep learning curve. Crucially, HIFU primarily ablates localized tumor tissue; it does not target systemic tumor dissemination via hematogenous spread, lymphatic metastasis, or distant seeding. Therefore, HIFU must be integrated with systemic therapies and serves as an effective adjunct to, rather than a replacement for, conventional cancer treatments.

## Conclusions

In conclusion, this case demonstrates that HIFU combined with chemotherapy is effective in treating recurrent endometrial stromal sarcoma. This combined treatment provides a new option for patients who refuse secondary surgery or cannot tolerate it. We hope that more clinical research and data will confirm its effectiveness and safety in the future, and further explore its mechanism of action in endometrial stromal sarcoma, especially its impact on immune function and the mechanism of action in increasing sensitivity and enhancing efficacy.

## Data Availability

The original contributions presented in the study are included in the article/**Supplementary Material**. Further inquiries can be directed to the corresponding authors.
