# Correction: High-intensity focused ultrasound as a combined approach for the treatment of recurrent low-grade endometrial stromal sarcoma: a case report and literature review

**DOI:** 10.3389/fonc.2026.1783476

**Published:** 2026-01-28

**Authors:** Huihui Chen, Xiaonan Shang, Yue Shen, Huajing Huang, Zebo Jiang, Qingyi Wang, Zhixing Cao, Peiyu Yan, Suying Xiao, Liangyu Chen, Donghui Huang, Min Kang

**Affiliations:** 1 Faculty of Chinese Medicine, Macau University of Science and Technology, Macao, Macao SAR, China; 2 Zhuhai Hospital of Integrated Traditional Chinese Western Medicine, Zhuhai, Guangdong, China; 3 Northeastern University, Boston, MA, United States; 4 Zhuhai People’s Hospital, Zhuhai, Guangdong, China; 5 State Key Laboratory of Quality Research in Chinese Medicines, Macao, Macao SAR, China; 6 Macau University of Science and Technology Zhuhai MUST Science and Technology Research Institute, Macao, Macao SAR, China

**Keywords:** LGESS, HIFU, tumor recurrence, combination therapy, case report

Author Zebo Jiang was erroneously spelled as Zhebo Jiang.

The original version of this article has been updated.

